# Endonasal Endoscopic Transsphenoidal Resection of Tuberculum Sella Meningioma with Anterior Cerebral Artery Encasement

**DOI:** 10.7759/cureus.311

**Published:** 2015-08-25

**Authors:** Sivashanmugam Dhandapani, Hazem M Negm, Salomon Cohen, Vijay K Anand, Theodore H Schwartz

**Affiliations:** 1 Department of Neurosurgery, Postgraduate Institute of Medical Education and Research, Chandigarh; 2 Department of Neurosurgery, Menoufia University; 3 Department of Neurosurgery, National Institute of Neurology and Neurosurgery, Manuel Velasco Suárez; 4 Department of Otorhinolaryngology, Weill Cornell Medical College, New York Presbyterian Hospital, New York; 5 Weill Cornell Brain and Spine Center, Weill Cornell Medical College, New York Presbyterian Hospital, New York

**Keywords:** endonasal endoscopy, tuberculum sella meningioma, anterior cerebral artery encasement, arachnoid plane, transtuberculum/transplanum approach

## Abstract

Anterior cerebral artery (ACA) encasement is often considered a contraindication for an endonasal endoscopic transsphenoidal approach. We report a patient with a tuberculum sella meningioma with ACA encasement, in whom a gross total excision was achieved through an endonasal endoscopic transsphenoidal transtuberculum, transplanum approach. The tumor was sharply dissected along the left ACA using meticulous bimanual sharp dissection after internal decompression. Moreover, the medial optic canals were opened and the optic nerves decompressed. A gasket seal closure with a nasoseptal flap was performed, and the patient was discharged on postoperative day four with improved vision. This case highlights the ability to remove planum and tuberculum meningiomas with vascular encasement through an endonasal endoscopic approach with the potential for safe vascular dissection. The absence of luminal narrowing can be used to assure the likelihood of a safe arachnoid plane.

## Introduction

Tuberculum sellae and planum meningiomas have been traditionally removed through a transcranial approach, which has been proposed to provide a complete visualization of the dural tail and the ability to use a microdissection technique to release encapsulated vessels and to open the optic canals. More recently, a minimal access endonasal endoscopic approach (EEA) has been utilized by select surgeons at specialized centers to remove these tumors and avoid a skin incision, large bone opening, temporalis muscle dissection, and brain retraction. The EEA offers other advantages, such as the complete removal of the hyperostotic bone, early devascularization of tumor, minimization of brain and optic nerve manipulation, feasibility of early bilateral optic canal decompression, and an improved view of tumor invading into the diaphragma sella and sella [1-5]. However, vascular encasement of the anterior cerebral artery (ACA) complex has been thought to be a contraindication to EEA based on the presumed technical challenge and the more limited maneuverability provided by the EEA [2, 6]. Here, we report a patient with a tuberculum sellae meningioma with encasement of the left ACA who underwent gross total excision of the tumor using the EEA and discuss the technical aspects of achieving endonasal vascular decompression.

## Technical report

**Figure 1:**
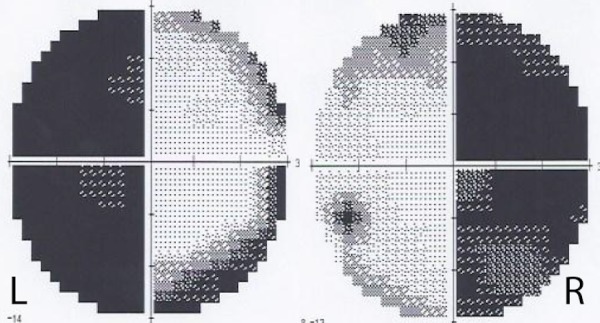
Baseline Visual Field of the Patient

A 46-year-old female presented with an insidious headache, progressive bitemporal hemianopia and occasional dizziness (Figure 1). An informed patient consent was obtained for this patient's treatment. No identifying patient information was used in this report.

**Figure 2:**
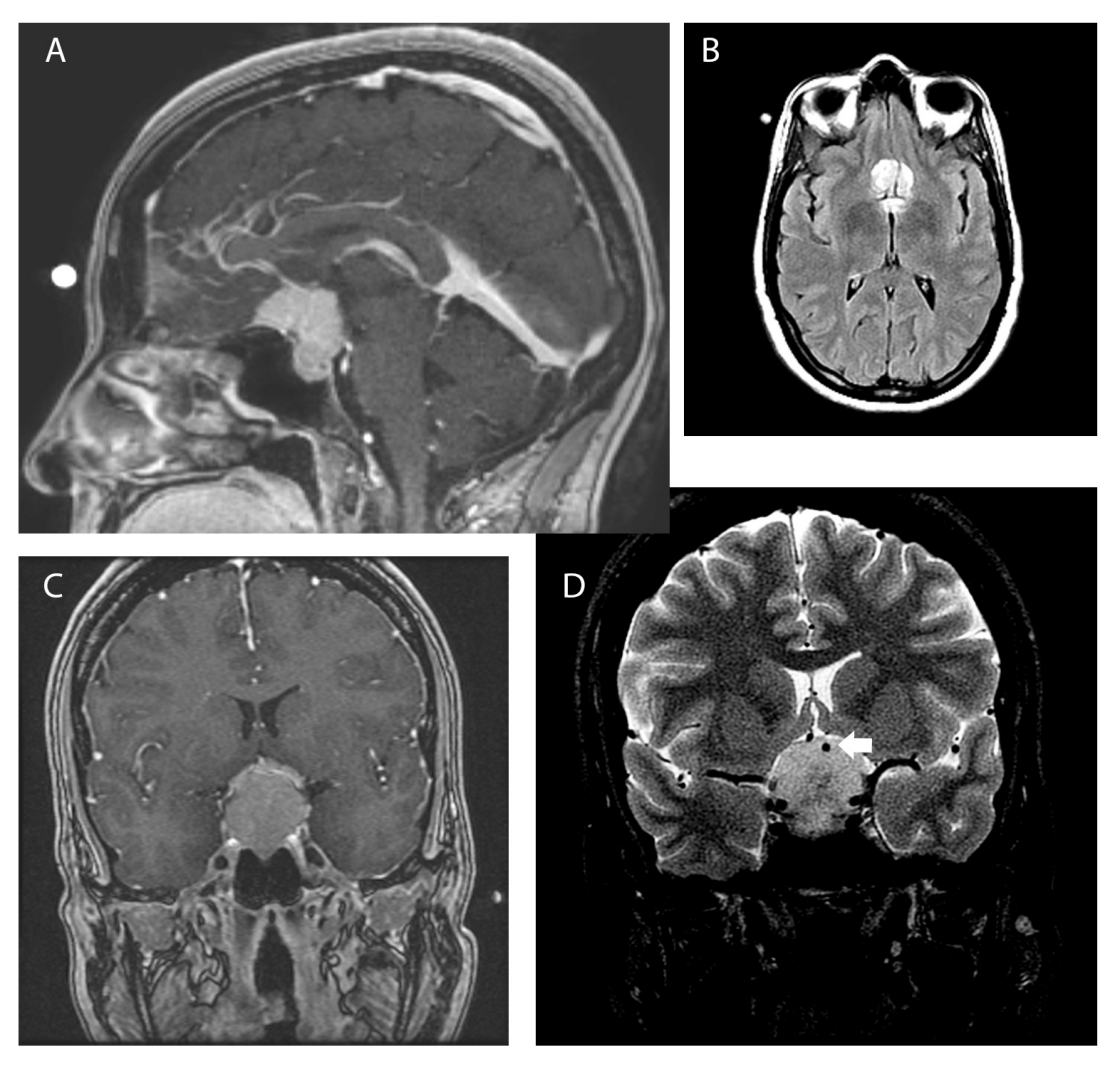
Pre-op Radiology

MRI showed a 3.2 cm extra-axial lesion centered on the tuberculum sella, hyperintense in T2/FLAIR, with intense homogeneous enhancement, and a dural tail suggestive of a tuberculum sellae meningioma (Figure 2).

MRI clearly showed a complete encasement of the left ACA (A1-A2 junction) as well as bilateral optic canal invasion (Figure 2). Needless to say, there was no “cortical cuff” between the tumor and the vessels. There were no signal changes in the adjacent brain that would indicate pial invasion.

**Figure 3:**
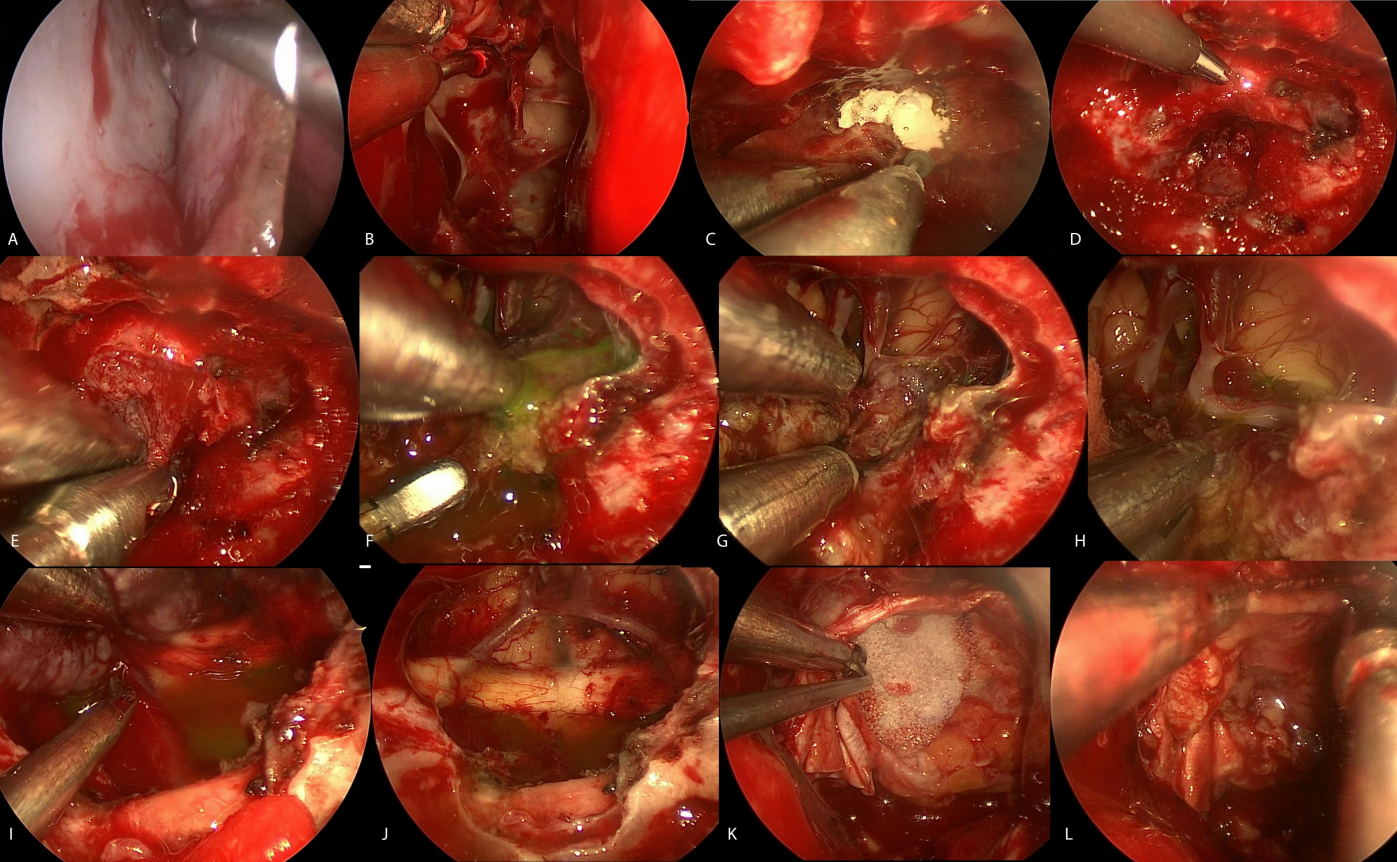
Intra-op Images

The general technique of EEA resection of tuberculum and planum meningiomas has been described previously [1, 7-8]. In brief, a lumbar drain is placed followed by an endoscopic endonasal approach to the sellar region. A nasoseptal flap is harvested (Figure 3A) and a wide sphenoidotomy performed (Figure 3B). The tuberculum and planum were removed with a diamond drill and Kerrison rongeur. In that way, the anterior extent of the tumor was exposed and the lateral exposure incorporated both optic canals (Figure 3C). The dural base was cauterized fully with bipolar and opened in the center (Figure 3D). The tumor was internally decompressed with suction, ring curettes, and the Elliquence (Elliquence, LLC, Baldwin, New York) (Figure 3E), and fluorescein dye was used to identify the subarachnoid plane. The superior aspect of the tumor was first removed until the A2 branches were identified. These were followed back to the encased left A2 and AComm. Using sharp dissection, the arachnoid plane was created around the vessels until the tumor could be rolled forward (Figure 3F-H). The medical optic canals were opened bilaterally, and the tumor was removed to fully decompress the optic nerves (Figure 3H). The dural attachment was fully excised following coagulation (Figure 3I). The defect was closed with a “gasket seal” technique, in which an onlay of fascia lata is held in place with a buttress of Medpor^©^ (Stryker, Kalamazoo, Michigan) wedged into the bone defect with care taken to avoid compressing the optic nerves (Figure 3K). The nasoseptal flap was placed over this construct all around (Figure 3L). A lumbar drain was left in place for 24 hours.

**Video 1:** Surgical Video

**Figure 4:**
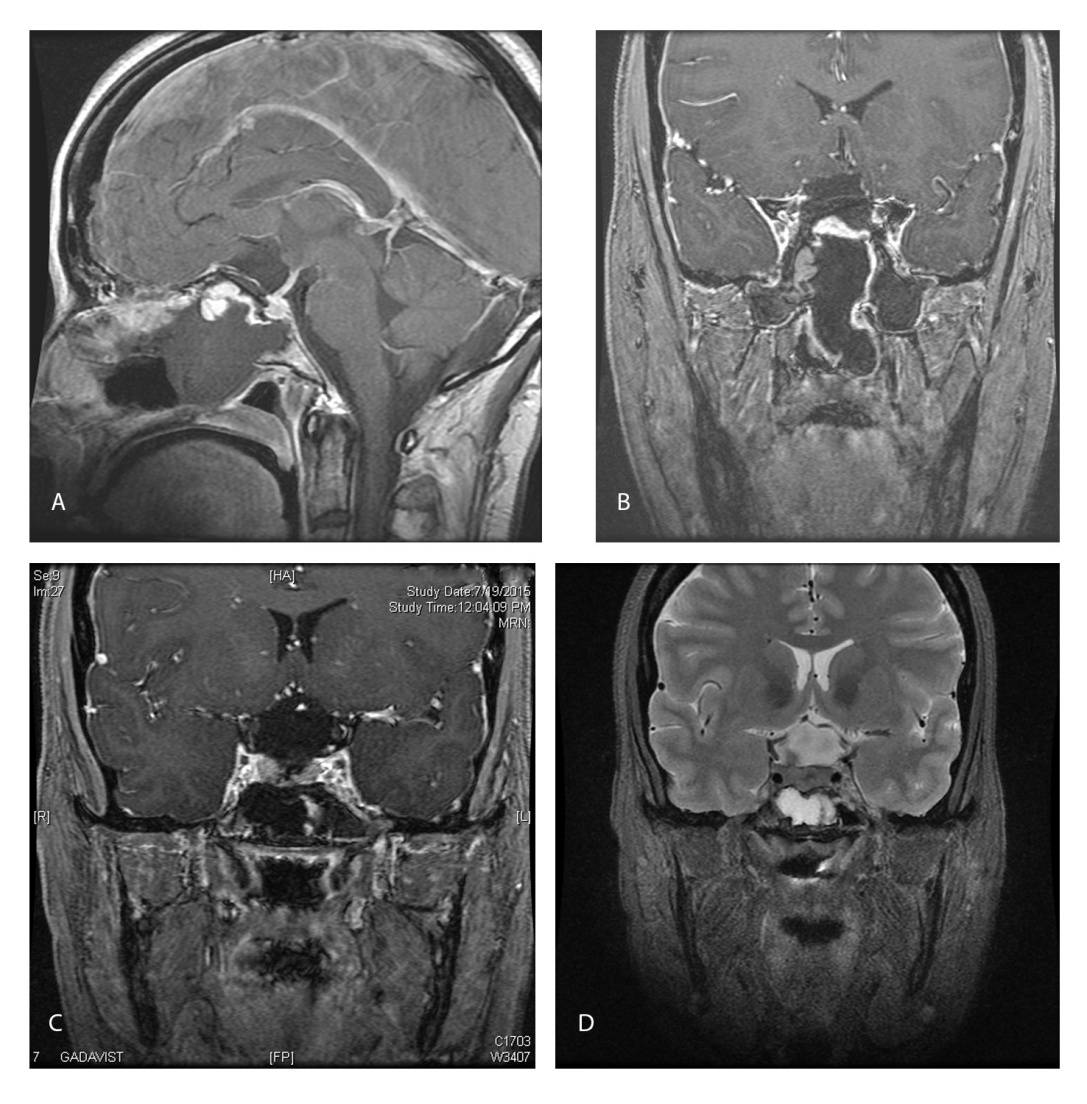
Postop Radiology

Postoperatively, the patient had improved vision. MRI (Figure 4) showed a gross total excision of the tumor with bilateral ACAs floating in subarachnoid space, complete decompression of both optic canals, and the vascularized nasoseptal flap in-situ.

## Discussion

Tuberculum sellae meningiomas arising from the tuberculum sellae, chiasmatic sulcus, limbus sphenoidale, and diaphragm sellae are challenging lesions because of their proximity to many critical structures, such as visual pathway, hypothalamus, stalk, and AComm complex. Traditionally managed by transcranial surgery [9], these have recently been managed by EEA, which offers several advantages [1-6, 10]. One presumed contraindication has been the absence of a “cortical cuff” separating the tumor from adjacent vessels as well as vascular encasement [2, 6, 10-11]. In this case report, we demonstrate the ability to dissect meningiomas off the ACA even in the face of complete encasement using standard bimanual microdissection technique through a minimal access EEA.

The involvement of adjacent vessels by skull base meningiomas is well known. Ishikawa, et al. had classified vessel involvement by meningiomas into engulfment and encasement [12]. Partial involvement, probably due to the lobularity, was termed “engulfment”, while complete involvement was termed “encasement”. The other most important factor determining total resectability of these tumors is probably the presence of intact arachnoid membranes between the tumor and vessels [13]. While tumors may violate the layers of vessels close to the dura (especially para-clinoidal ICA), causing narrowing of the lumen, their involvement of vessels distant from the dura may be extra-arachnoidal [12-13]. Hence, tuberculum sellae meningioma involvement of the ACA junction without luminal narrowing, whether engulfment or encasement, is likely to be extra-arachnoidal.

The potential injury to the ACA junction during EEA is more often due to the avulsion of perforators from ACA [4, 7, 10-11]. This can be avoided by meticulous internal decompression and gentle bimanual extra-arachnoidal sharp dissection without traction on the perforators and main vessels. As we had mentioned under the technique, the cuff of the tumor encasing the ACA is opened up with this technique, so that safe gross total excision can be achieved.

Koutourousiou, et al. had reported vascular encasement in 19 out of 75 suprasellar meningiomas, and only six out of 19 had gross total excision [11]. However, further typing based on their location, an involvement of the ACA or ICA, with or without luminal narrowing, were not separately reviewed. Khan, et al. had noted the presence of a “cortical cuff” to be not as significant a predictor of outcome as was careful case selection and surgical experience [10]. As experience with EEA technique proliferates and equipment advances, previously described contraindications will be overcome. In this report, we find and show that ACA involvement without luminal narrowing is likely to be extra-arachnoidal, thereby allowing gross total tumor excision with an EEA transsphenoidal transtuberculum approach. 

## Conclusions

In the absence of luminal narrowing, meningiomas with vascular encasement can be removed using minimal access EEA, provided the surgeon has sufficient experience and appropriate equipment. ACA involvement, even encasement, without narrowing should not be a contraindication to EEA.
